# Temporal Dynamics of Cloacal Microbiota in Adult Laying Chickens With and Without Access to an Outdoor Range

**DOI:** 10.3389/fmicb.2020.626713

**Published:** 2021-01-28

**Authors:** Janneke Schreuder, Francisca C. Velkers, Alex Bossers, Ruth J. Bouwstra, Willem F. de Boer, Pim van Hooft, J. Arjan Stegeman, Stephanie D. Jurburg

**Affiliations:** ^1^Department Population Health Sciences, Faculty of Veterinary Medicine, Utrecht University, Utrecht, Netherlands; ^2^Department of Infection Biology, Wageningen Bioveterinary Research, Lelystad, Netherlands; ^3^GD Animal Health, Deventer, Netherlands; ^4^Wildlife Ecology and Conservation Group, Wageningen University and Research, Wageningen, Netherlands; ^5^German Centre for Integrative Biodiversity Research (iDiv), Halle-Jena-Leipzig, Leipzig, Germany

**Keywords:** poultry (chicken), cloacal microbiota, 16S rRNA gene amplicon sequencing, temporal dynamics, host microbiome, outdoor range

## Abstract

Associations between animal health and performance, and the host’s microbiota have been recently established. In poultry, changes in the intestinal microbiota have been linked to housing conditions and host development, but how the intestinal microbiota respond to environmental changes under farm conditions is less well understood. To gain insight into the microbial responses following a change in the host’s immediate environment, we monitored four indoor flocks of adult laying chickens three times over 16 weeks, during which two flocks were given access to an outdoor range, and two were kept indoors. To assess changes in the chickens’ microbiota over time, we collected cloacal swabs of 10 hens per flock and performed 16S rRNA gene amplicon sequencing. The poultry house (i.e., the stable in which flocks were housed) and sampling time explained 9.2 and 4.4% of the variation in the microbial community composition of the flocks, respectively. Remarkably, access to an outdoor range had no detectable effect on microbial community composition, the variability of microbiota among chickens of the same flock, or microbiota richness, but the microbiota of outdoor flocks became more even over time. Fluctuations in the composition of the microbiota over time within each poultry house were mainly driven by turnover in rare, rather than dominant, taxa and were unique for each flock. We identified 16 amplicon sequence variants that were differentially abundant over time between indoor and outdoor housed chickens, however none were consistently higher or lower across all chickens of one housing type over time. Our study shows that cloacal microbiota community composition in adult layers is stable following a sudden change in environment, and that temporal fluctuations are unique to each flock. By exploring microbiota of adult poultry flocks within commercial settings, our study sheds light on how the chickens’ immediate environment affects the microbiota composition.

## Introduction

The digestive tract of chickens is colonized by complex microbial communities, which play important roles in their overall health and performance (Yegani and Korver, 2008; Ducatelle et al., 2015; Kogut, 2019). Changes in the chickens’ microbiota have been linked to many factors (Kers et al., 2018), including host related factors such as age (Cox et al., 2014; Videnska et al., 2014; Jurburg et al., 2019; Ngunjiri et al., 2019) and breed (Schokker et al., 2015; Richards et al., 2019). Outside of the host, differences in climate, soil, litter, and feed affect the host’s exposure to other microbes, which may colonize the animal’s intestinal tract (Björk et al., 2019). Indeed, under controlled settings, housing conditions have been found to modulate the chickens’ microbiota (Hubert et al., 2019; Kers et al., 2019). How the gut microbiota responds over time to changes in the housing environment under standard farm conditions is less well understood, however.

In commercial settings, layers may be restricted to indoor housing, or may have access to an outdoor range. Layers housed in free-range environments have different microbial community compositions and higher diversity than indoor housed layers (Xu et al., 2016; Chen et al., 2018; Hubert et al., 2019). However, in these studies the effect of access to a free range was compared between caged and free-range chickens in semi-experimental setups (Xu et al., 2016; Chen et al., 2018; Hubert et al., 2019). Furthermore, chickens were given access to the outdoor range during the rearing period (6–11 weeks of age, Xu et al., 2016; Chen et al., 2018). An increasing amount of commercial layer flocks are kept in aviary systems rather than in cages (Miao et al., 2005), in which the layers in free-range or organic systems are given access to an outdoor range after the rearing period (approximately 17 weeks of age). Previous research showed that access to an outdoor range only explained limited variation in the community composition in a cross-sectional study (Schreuder et al., 2020). However, this study sampled animals only once after long-term acclimation and it was not possible to determine whether the microbiota had been affected by outdoor exposure and recovered to their original composition over time, i.e., resilient, or whether the microbiota were resistant to outdoor range exposure, i.e., resistant (Sommer et al., 2017). These temporal dynamics and the immediate effects of exposure to a new environment remain poorly understood.

Microbial communities exhibit complex, non-linear temporal dynamics, especially during host development (Jurburg et al., 2019; Kers et al., 2020). Understanding how the hosts’ microbiota respond to environmental fluctuations requires temporal monitoring in order to detect changes in the microbial community over time, following exposure to new conditions. To date, a limited number of studies have explored the temporal dynamics in the gut microbiota of layers (Videnska et al., 2014; Ballou et al., 2016; Han et al., 2016; Polansky et al., 2016; Ngunjiri et al., 2019). Most studies focus on young layers (aged 0–8 weeks), and are performed under experimental conditions (Ballou et al., 2016; Polansky et al., 2016; Han et al., 2017). However, adult chickens have fully developed microbiota, which are more stable than microbial communities of young layers (Videnska et al., 2014; Ngunjiri et al., 2019). It has been proposed that as an animal ages, the host’s influence on microbial selection increases due to physiochemical maturation of the gut and the ability of the host to curate its microbiota (Björk et al., 2019), likely making the microbiota of adult layers less prone to external perturbations or changes (Schreuder et al., 2019).

It is essential to understand how the gut microbiota of commercial animals respond to environmental changes to guarantee their health in the face of unforeseen events, such as disease outbreaks. To examine the extent to which sudden environmental changes affect the gut microbiota of adult layers in commercial setting, we monitored the cloacal microbiota of four flocks of laying hens over 4 months following the lifting of mandatory indoor confinement regulations, which was a unique opportunity to study the effects of the outdoor range access on the microbiota of commercial chickens over time. Over a 16 weeks period, we sampled 10 chickens per flock three times in 8 weeks intervals. We hypothesized that as layers accessed an outdoor range, they would be more exposed to alternative food sources and novel environmental microbes, and microbial richness would increase over time in outdoor chickens. If the colonization of novel microbes occurred stochastically (i.e., randomly), we also expected the microbiota of outdoor flocks to become more variable between outdoor chickens than indoor chickens.

## Materials and Methods

### Study Design

**FIGURE 1:**
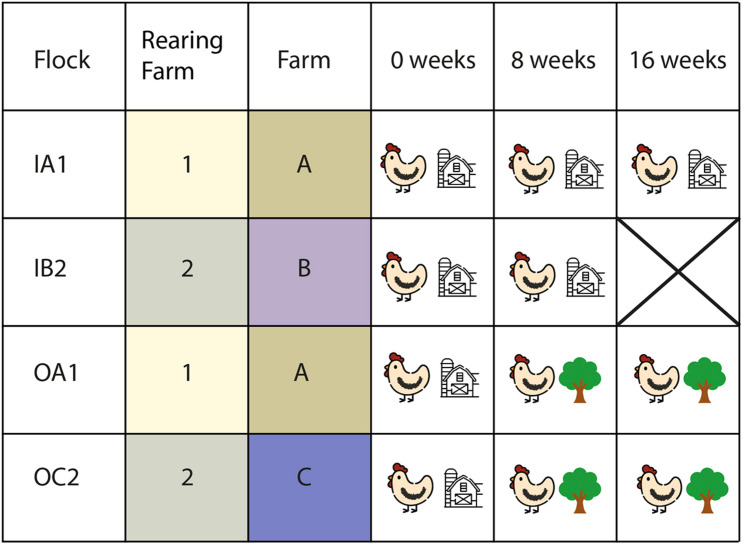
Study design. Four flocks were sampled three times (0, 8, and 16 weeks after the study began) each. Two indoor flocks (IA and IB) and two outdoor flocks (OA and OC) were sampled. Flock IB2 was not sampled on week 16. Week 0 began 1–2 days after the lift of mandatory indoor housing ban of all layer flocks and none of the layer flocks had access to the outdoor range prior to that moment. Flocks IA1 and OA1 were located on the same farm (1), originated from the same rearing flock (A) and were of the same age (33 weeks at the start of sampling). Flocks IB2 and OC2 were located on two different farms (2 and 3), originated from the same rearing flock (B) and were of the same age (24 weeks at start of sampling). Cloacal swabs of 10 chickens per flock were collected at each sampling time.

Four commercial flocks of laying hens (Dekalb White) were selected for this study: two layer flocks with access to an outdoor range and two flocks without access to an outdoor range (Figure 1). To minimize potential variation in the microbiota composition due to rearing and other environmental factors, outdoor and indoor flocks were selected based on the rearing farm of origin, numbers 1 and 2, respectively (Figure 1). The sampled flocks were kept in separate poultry houses, which were located on three different poultry farms: indoor (IA1) and outdoor flock 1 (OA1) were located on the same farm, indoor (IB2), and outdoor flock 2 (OC2) were located on two different farms (Figure 1). Flocks IA1 and OA1 were 33 weeks old at the start of the sampling, and flocks IB2 and OC2 were 24 weeks old. All flocks were well-producing and healthy based on regular veterinary inspections during the study, and had not been treated with antibiotics on the layer farm.

None of the layers from the indoor or outdoor flocks had access to the outdoor range prior to the start of this study due to the mandatory indoor confinement measures, which were instated because of HPAI outbreaks in the winter of 2016–2017. All flocks were sampled three times in 2017: the first sampling took place 1–2 days after the lift of mandatory indoor confinement at the end of April 2017; and the second and third sampling rounds took place 8 and 16 weeks after the lifting date, respectively (Figure 1). During each sampling round, all flocks were sampled in the same week, to avoid short term weather effects. We did not sample flock IB2 on the third sampling round, because these chickens were in the process of forced molting at that time, in order to reduce fipronil contamination during the fipronil affair in the Netherlands (Sok et al., 2020). Molting was induced by feed deprivation, and feed deprivation has major impact on the gut microbiota composition (Dunkley et al., 2007), making the samples of the chickens of flock IB2 unsuitable for our study at the time of the third sampling.

Both indoor and outdoor layer flocks were kept in cage-free aviary systems with a maximum stocking density of nine chickens per m^2^, with one flock per house (Schouwenburg, 2019a). The hens of each flock were placed in the poultry house on the layer farm around 17 weeks of age. Flock IB2 had a different feed supplier than OA1, IA1, and OC2, but all flocks received a similar standard commercial feed for layers according to their age with a similar regime across farms, and no changes in the feed composition occurred during the period of the study. The laying hens in outdoor flocks had access to an outdoor range during the day with at least 4 m^2^ per hen according to standards of the Dutch quality assurance scheme, i.e., the Integrated Chain Control program, “IKB Egg” (Schouwenburg, 2019b). The hens had access to the outdoor range for 8 h per day on average (van Niekerk and Leenstra, 2016). Outdoor ranges were mostly open grass field with some trees, and bare soil directly around the poultry house, with drainage systems to prevent formation of rain puddles (van Niekerk and Leenstra, 2016).

### DNA Sampling, Extraction, and 16S rRNA Gene Amplicon Sequencing

At each sampling time, two cloacal dry swabs per chicken were collected from 50 laying hens per flock. The swabs were inserted deep into the cloaca to ensure we would collect enough fecal material. Wired panels, dividing the house in multiple subsections, were present in all houses, and an equal number of birds was randomly selected from each subsection within each flock. Samples were placed on ice immediately after collection and stored dry at −80°C within 5 h after collection.

Per flock and sampling time, swabs of 10 of the 50 sampled chickens were selected based on equal distribution across the poultry house. One swab of each chicken was used for analysis. Prior to analysis, each swab was visually assessed to ensure that sufficient fecal material was present for DNA extraction. DNA extraction and subsequent 16S rRNA gene amplicon sequencing were performed according to the protocol described in Schreuder et al. (2020). In each DNA isolation round, a negative control sample containing PBS was added to identify possible contamination from reagents, and DNA extracts were quantified with Invitrogen^TM^ Qubit^TM^ 3.0 Fluorometer and stored at −20°C for further processing. The V3–V4 region of the 16S rRNA gene was amplified in a PCR with the primers CVI_V3-forw CCTACGGGAGGCAGCAG and CVI_V4-rev GGACTACHVGGGTWTCT and amplified as previously described (Schreuder et al., 2019): step 1: 98°C for 2 min, step 2: 98°C for 10 s, step 3: 55°C for 30 s, and step 4: 72°C for 10 s, step 5: 72°C for 7 min. Steps 2–4 were repeated 25 times. Negative controls were included at each amplification round to confirm sterility of PCR reagents. PCR products were checked with gel electrophoresis, and PE300 sequencing was performed using a MiSeq sequencer (Illumina Inc., San Diego, CA). Negative controls from the DNA extraction did not contain any sequences and were discarded (*n* = 6). An additional 16S rRNA gene qPCR was performed on the DNA samples to quantify the amount of 16S rRNA gene DNA and identify samples of poor quality (Supplementary Table S1). The qPCR consisted of 40 runs with the same primers and protocol as for the 16S barcoding PCR. Samples which contained very low 16S rRNA gene DNA concentrations or low quality melting curves were excluded from the analysis (*n* = 10). The final dataset contained 100 samples. The number of samples per house for each sampling time ranged between 7 and 10 samples at each timepoint (Supplementary Table S1).

### Processing of Sequencing Data

All sequence data processing was performed in R 3.6.3 (R Core Team, 2013). The sequence reads were quality-filtered, primer-trimmed (35 nucleotides), error-corrected, dereplicated, merged into pseudoreads and chimera-filtered using the dada2 package (Callahan et al., 2016) using standard parameters [TruncLength = (240, 210), MinOverlap = 10 and maxEE = (2,2)], and reads were assigned with the SILVA v.132 classifier (Quast et al., 2012). The final dataset contained 100 samples, which were rarefied to 8,170 reads per sample (rarefy_even_depth, seed = 1) and a total of 2,839 amplicon sequence variants (ASVs) distributed over 347 genera.

### Statistical Analyses

All downstream analyses were performed in R (version 3.6.3) with the phyloseq (McMurdie and Holmes, 2013) and vegan (Oksanen et al., 2007) packages. We measured diversity as the number of observed ASVs in the rarefied samples and taxon evenness with Pielou’s index (Pielou, 1966). A linear mixed effects model was fitted to both diversity measures, with poultry house as a random effect and sampling time and housing type as fixed effects using the lme4 package (Bates et al., 2015). Bray and Curtis (1957) and Sørensen (1948) dissimilarities were used to evaluate differences in community structure between the layers on Hellinger-transformed abundances. Community composition was visualized with principal coordinates analyses (PCoA) of Bray-Curtis and Sørensen dissimilarities. Differences between the microbiota composition of layers were examined for each factor using the adonis function. Variance in community composition within a flock was evaluated as the Bray–Curtis and Sørensen pairwise distances between flock members. To assess the contribution of each factor to the observed variation in the microbiota composition, we performed a distance-based variation partitioning (Borcard et al., 1992) and distance-based redundancy analysis (dbRDA) using Bray–Curtis dissimilarities (Anderson, 2001). We included housing type (indoor and outdoor layers, HousingType), poultry house (stable in which flocks were housed) and sampling time (SamplingTime) as explanatory variables. Feed, age, farm, and rearing farm were nested within poultry house (Figure 1 and Supplementary Table S1), and thus were not included. Model selection for dbRDA was performed with forward selection based on Akaike’s Information Criterion (AIC), with the lowest AIC indicating the best fit (Blanchet et al., 2008).

To visualize the number of taxa that were shared between poultry houses across sampling times, we used Venn-diagrams on rarefied data. In the Venn-diagrams, taxa were considered as rare when the relative abundance was < 0.01% across all samples. We used Wilcoxon rank-sum tests to check for differences in relative abundances of the 10 most abundant phyla and of genera with a relative abundance of at least 0.5% over time within each housing type. Unless otherwise indicated, results are expressed as mean ± *SD* throughout the manuscript. We used DESeq2 analysis (Love et al., 2014) on non-rarefied data to detect ASVs that were differentially abundant over time between indoor and outdoor housed chickens.

Figures made with ggplot2 and ggpubr packages.

## Results

### Microbial Community Composition

We evaluated the composition of the microbial community in the cloacal samples of all layers. At the phylum level, we observed similarities between the microbiotas of indoor and outdoor layers (Supplementary Figure S1), and no significant differences in the relative abundances of the 10 most abundant phyla were found. These 10 phyla constituted 99.8% of the community, across all samples. On average across all samples, the microbial communities were dominated by Firmicutes (63.7 ± 17.3%), Proteobacteria (13.4 ± 14.3%), and Fusobacteria (9.0 ± 15.4%; Supplementary Figure S1). At genus level, members of the genera Romboutsia (31.4 ± 22.3%), Gallibacterium (9.5 ± 12.1%), and Fusobacterium (8.9 ± 15.2%) were most abundant across all samples (Supplementary Figure S2).

**FIGURE 2:**
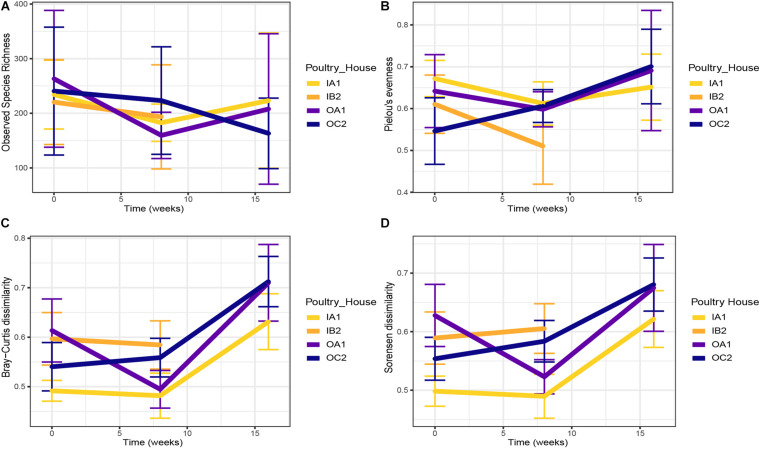
Temporal trends in observed species richness **(A)** and Pielou’s evenness **(B)** per poultry house at each sampling time. Pairwise Bray–Curtis **(C)** and Sørensen **(D)** dissimilarities between the cloacal microbiota of layers from poultry house at each sampling time. In C and D, greater values indicate higher dissimilarity. Means ± confidence interval are shown. Time is shown as weeks since first sampling.

We did not find temporal patterns in species richness in both indoor and outdoor housed chickens (Figure 2A). A modest, but significant increase in evenness was detected in chickens from outdoor houses over time (from 0.59 ± 0.12 at 0 weeks to 0.70 ± 0.13 at 16 weeks; *p* < 0.001; Figure 2B).

To analyze changes in community composition over time, we evaluated Bray–Curtis and Sørensen dissimilarities between chickens of each flock at each sampling time (Figures 2C,D). Although microbiota of chickens in outdoor flocks were more variable than those of indoor flocks on average, this was not significant, and the variation did not significantly increase over time (Figures 2C,D). Across all samples, variation between chickens from each poultry house had increased at 16 weeks compared to the first sampling (*p* < 0.001).

**FIGURE 3:**
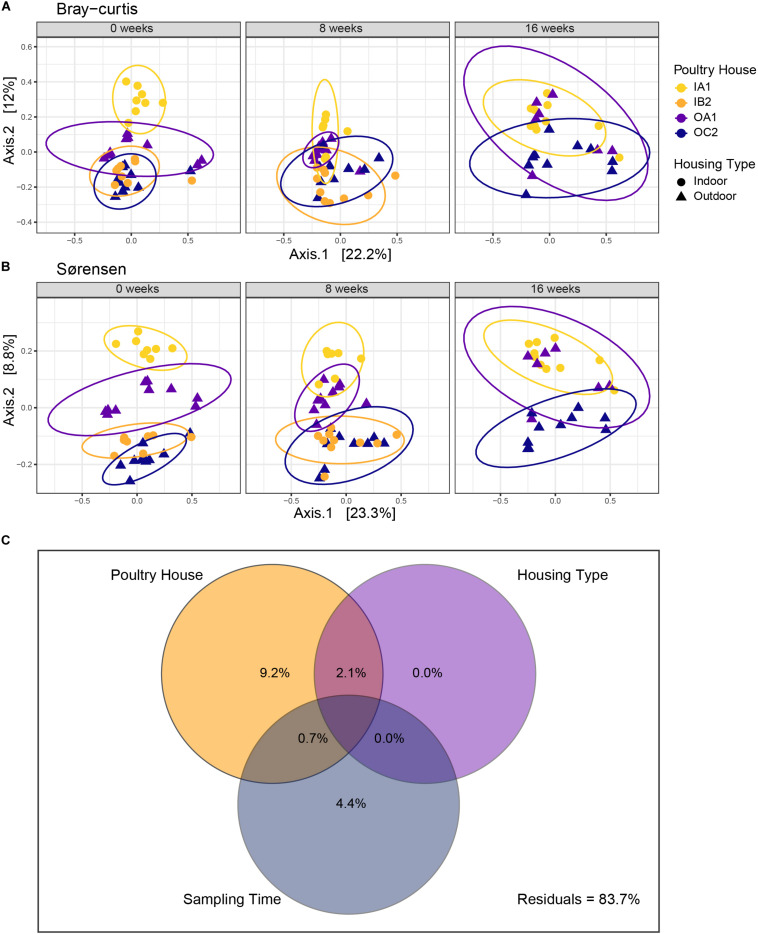
Principal coordinate analysis (PCoA) of Bray–Curtis **(A)** and Sørensen **(B)** dissimilarities. Each PCoA is faceted per sampling time, with ellipses encircling poultry houses. Each symbol represents an individual chicken. Time is shown as weeks since first sampling. **(C)** Distance-based partitioning of Bray–Curtis dissimilarities with poultry house, housing type and sampling time as explanatory variables. Adjusted *R*^2^ are shown in each circle. The model explains 14.8% of variation in community composition overall.

For both dissimilarity measures, microbial communities clustered according to poultry house (Figure 3), which explained most of the variance in the community (Bray–Curtis *R*^2^ = 14.5%, adonis, *p* < 0.001; Sørensen *R*^2^ = 14.5%, adonis, *p* < 0.001; Table 1). Sampling time (Bray–Curtis *R*^2^ = 2.97%, adonis, *p* = 0.013, Sørensen *R*^2^ = 3.75%, adonis, *p* < 0.001) and housing type (Bray–Curtis *R*^2^ = 2.91%, adonis, *p* = 0.001; Sørensen *R*^2^ = 3.41%, adonis, *p* < 0.001) explained limited variation, but were significant for both dissimilarity measures (Table 1).

**TABLE 1:** Overview of explained variation in community composition by individual factor as tested with a PERMANOVA-like adonis.

Factor	Dissimilarity	*R*^2^	F. Model	*p*	F Dis
	measure	(adonis)			
Time	BC	2.97	2.98	0.0013	0.01
	Sørensen	3.75	3.82	< 0.001	0.11
Housing type	BC	2.91	2.94	0.001	0.78
	Sørensen	3.41	3.46	< 0.001	0.19
Poultry house	BC	14.49	5.42	< 0.001	0.68
	Sørensen	14.48	5.42	< 0.001	0.26
Farm	BC	11.81	6.50	< 0.001	0.65
	Sørensen	11.81	6.50	< 0.001	0.48
Rearing farm	BC	8.42	9.01	< 0.001	0.46
	Sørensen	7.43	7.87	< 0.001	0.14

**Both Bray–Curtis and Sørensen dissimilarity were used. R^2^ = Percentage of the variation between chickens explained. Significance was tested with 9,999 permutations.**

**FIGURE 4:**
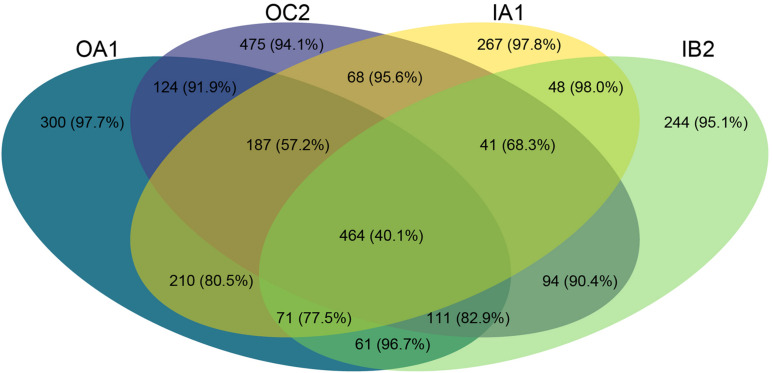
Venn diagram depicting the number of shared ASVs per poultry house across all timepoints. In parentheses, the percentage of these ASVs which are rare (relative abundance of < 0.01%) are shown.

To further disentangle the effects of poultry house, sampling time and housing type, we performed a distance-based variance partitioning using Bray–Curtis dissimilarities (Figure 4). Poultry house explained most of the variation in community composition (9.2% *R*^2^_*a*__*dj*_) and sampling time explained 4.4% of the variation (*R*^2^_*a*__*dj*_). In contrast, housing type alone did not explain any variation. This was further supported by a dbRDA (Supplementary Figure S2). Model selection supported a model with both poultry house and sampling time (AIC = 301.38) compared to a full model, with housing type and the interaction between housing type and sampling time (AIC = 304.64). Poultry house (*p* = 0.005) and sampling time (*p* = 0.005) were both significant in this most parsimonious model (Supplementary Figure S2).

### Differential Abundance of Individual Taxa Over Time

**FIGURE 5:**
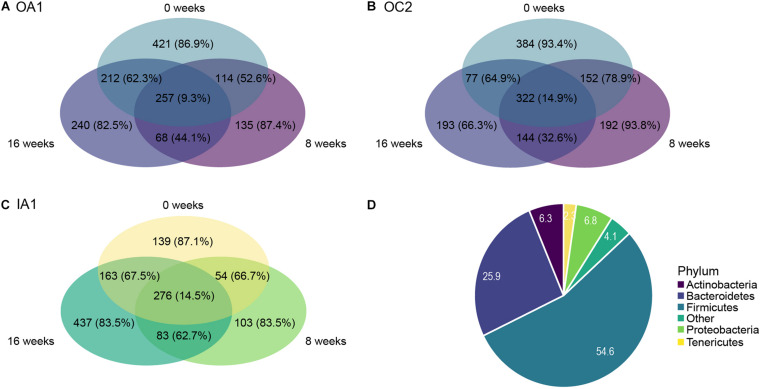
Venn diagrams of each poultry house [**(A)** OA1; **(B)** OC2; **(C)** IA1] showing the number of ASVs shared across all timepoints or unique to a single sampling time. Poultry house IB2 was excluded. Number of ASVs are shown for each compartment, and the percentage of rare taxa (ASVs with relative abundance of < 0.01%) are shown in parentheses. **(D)** Relative abundances (%) of five most abundant phyla within the subset of rare ASVs (relative abundance < 0.01% across all samples).

We identified ASVs that were shared by the different poultry houses over time (Figure 4). ASVs that were shared between all poultry houses had a lower percentage of rare taxa (40.1%) than ASVs that were unique to a poultry house (between 94.1 and 98.0%, Figure 4). Each poultry house had a similar number of shared taxa between all sampling times (between 257 and 322 ASVs, with 9.3–14.9% rare ASVs), whereas the amount of unique taxa to a sampling time varied between 103 and 437 ASVs, but the percentage of rare ASVs was similar at each sampling time ranging between 66.3 and 93.8% (Figures 5A–C). Most rare ASVs belonged to the phyla Firmicutes (54.6%) and Bacteroidetes (25.9%, Figure 5D).

**FIGURE 6:**
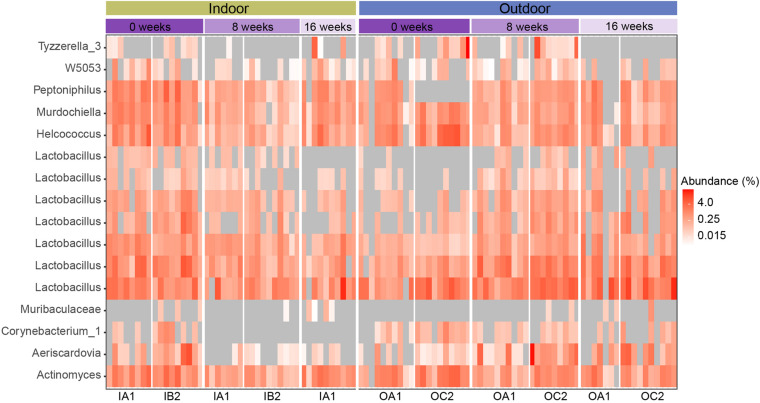
Heatmap of the relative abundance of 16 differentially abundant ASVs that were significantly different over time between indoor and outdoor housed chickens (DESeq2, fdr *p* < 0.01%). Samples are ordered by poultry house for each sampling time and divided by housing type (indoor vs. outdoor). Each box represents the relative abundance of an ASV in an individual chicken. 0 values are shown as gray boxes.

DESeq2 analysis was performed to determine if specific ASVs were differentially abundant over time between indoor and outdoor housed chickens. We compared a full model with factors: HousingType + SamplingTime + HousingType:SamplingTime to a reduced model with factors HousingType + SamplingTime, and identified 16 ASVs with differential responses (Supplementary Figure S4). These 16 ASVs belonged to nine genera in two phyla, Firmicutes and Actinobacteria. Most ASVs (*n* = 8) belonged to the genus Lactobacillus (Figure 6). The 16 ASVs had an average relative abundance of 0.60 ± 0.65% across all samples, but none of the ASVs showed a consistent increase or decrease in all chickens of one housing type over time (Figure 5 and Supplementary Table S2). The genus Lactobacillus also fluctuated significantly over time in outdoor housed chickens (*p* < 0.001, Kruskal–Wallis test), but not in samples from indoor housed chickens (*p* > 0.001, Kruskal–Wallis test). Furthermore, genera Akkermansia and Aeriscardovia (*p* < 0.001, Kruskal–Wallis test both) fluctuated significantly over time in outdoor chickens, but not in indoor housed chickens (Supplementary Table S3).

## Discussion

Many factors in the immediate environment of the chicken can influence the microbiota community composition (Kers et al., 2018). In this temporal study in commercial laying hens, we found that of the variables measured, poultry house explained most variation in community composition in the flocks’ microbiota (9.2%), whereas access to an outdoor range (housing type) did not explain any of variation in the microbial community. Some temporal effects were found, but the proportion of variation explained by time of sampling (4.4%) was comparatively smaller than that of poultry house. At the level of community diversity, flocks which were allowed into the outdoor range did not become more variable or more species-rich over time, and the chickens’ microbiota showed a modest but significant increase in evenness over time in outdoor flocks, but not in indoor flocks. The latter was not accompanied by changes in species richness over time, which indicates that the increase in evenness over time in outdoor layers did not result from the colonization of more species in the chickens’ microbiota, but rather from a shift in abundances. Abundances of several ASVs were found to fluctuate differently between indoor and outdoor layers over time. However, none of the ASVs showed a consistent increase or decrease in all chickens of one housing type over time. Previous research found a slightly higher variation in community composition in indoor flocks relative to outdoor flocks, but also found large differences in variation between poultry houses from the same housing type (Schreuder et al., 2020). In this study, the poultry house also was the most important driver of community composition, and outdoor range access only had a modest effect on the microbiota community of chickens across eight separate flocks (Schreuder et al., 2020). The results of the current study further highlight that the environment of the poultry house is an important driver for community composition, even over time.

We found that differences in microbial communities over time between layers within each flock were most likely driven by the replacement of rare taxa between sampling times within a poultry house. Indeed, most of the taxa, between 66.3 and 93.8%, at each sampling time were rare, and 94.1–97.8% of taxa that were unique to a poultry house, were also rare. Moreover, no difference in explained variation was found when communities were weighted by their relative abundances (Bray–Curtis dissimilarity) compared to using presence/absence data (Sørensen dissimilarity), indicating that taxon abundance was likely less relevant in differentiating these communities. Costa et al. (2017) also found that treatment with different antimicrobials resulted in changes in community membership of cecal microbiota of broilers, but not in community structure, suggesting that the antimicrobials had a greater impact on rare taxa, rather than on dominant ones. These findings indicate that temporal fluctuations are unique to each flock within each poultry house and support the need to learn more about the functional role of rare bacteria, and the need for techniques which focus on analyses of active bacteria (i.e., metatranscriptomics).

The strong influence of poultry house on the microbiota suggests that the living environment of the chicken is important in shaping the hens’ microbiota, however we found no effect of moving outdoors. One explanation for this phenomenon and the relatively small effect of sampling time on the community composition compared to previous research (Jurburg et al., 2019; Kers et al., 2019), is the developmental stage of the chickens studied. Layers of flocks in this study were adult chickens of either 24 or 33 weeks old at the first time of sampling. To date, most temporal studies in chickens looked at the temporal dynamics of young chickens and thus at changes in the primary environment of the host as a result of the host’s development (Cox et al., 2014; Oakley and Kogut, 2016; Jurburg et al., 2019; Kers et al., 2019; Richards et al., 2019). Here, we studied the effect of temporal changes in a secondary environment in adult layers (i.e., indoor or outdoor range), where the effect of the outdoor range was likely dampened by the adults’ host homeostatic responses. As an animal host ages, its influence on microbial selection in the development of the intestinal microbiota increases (Björk et al., 2019). Indeed, layers above the age of 25 weeks (Ngunjiri et al., 2019) or 28 weeks (Videnska et al., 2014) reach an adult microbial equilibrium (Videnska et al., 2014). It is likely that in our case the chicken microbiota was more plastic at an earlier stage, as we still see a strong effect of the rearing farm on the chickens microbiota in this study (Table 1). In the Dutch table egg production system, groups of laying hens reared together in one rearing farm are transported to the poultry houses of the final layer farm at the age of 17–18 weeks. By the time the layers were allowed outside in our study, the layers’ intestinal microbiota had likely already reached a stable equilibrium, which is less prone to perturbations (Schreuder et al., 2019). A well-developed intestinal microbiota community protects the host by creating gastrointestinal resistant environments, which help prevent external microbiota from colonizing, i.e., resistant (Lawley and Walker, 2013; Han et al., 2017), and is characterized by a capacity for self-regeneration after an external perturbation, i.e., resilience (Lozupone et al., 2012; Sommer et al., 2017). In previous research, it was not possible to determine whether the microbiota of adult layers were resistant or resilient after exposure to an outdoor range, because the temporal changes weren’t taken into account. The current study indicates that the microbiota of these adults layers was likely resistant rather than resilient.

Alternatively, the limited effect of the outdoor range on the chickens’ microbiota may occur if the chickens only made limited use of the outdoor range, despite having access. The effects of access to an outdoor range in previous studies (Xu et al., 2016; Chen et al., 2018; Hubert et al., 2019; Ocejo et al., 2019) have been related to greater substrate diversity and intake of fibrous feedstuff (Xu et al., 2016), as well as exposure to more abundant microbiota from the outdoor environment (Hubert et al., 2019). However, likely only a small proportion of the hens in the outdoor flocks of our study used the outdoor range extensively. Previous research estimated that only 3–15% of layers in large commercial flocks (> 10,000 layers) used the outdoor range (Bestman and Wagenaar, 2003; Hegelund et al., 2005), with individual hens using the range differently, of which many did not enter the free-range every day (Gebhardt-Henrich et al., 2014). Nevertheless, chickens that do not go outdoors themselves could indirectly become affected by the altered microbiota of their flock mates that do go outside, as these also defecate indoors. Humans and animals that are housed together are known to exchange microbiota (Song et al., 2013; Schloss et al., 2014), and this effect may be enhanced for coprophagic animals, including chickens (Kers et al., 2018; von Waldburg-Zeil et al., 2019). However, with a rather stable microbiota community, the small changes in the chickens that go outdoors are also less likely to affect the stable microbiota community of the chickens remaining indoors. Furthermore, other studies have shown that chickens tend to use the area immediately outside the poultry house most (Hegelund et al., 2005; Bestman, 2017), resulting in trampled vegetation and hence, lower availability of fibrous feedstuff. Both the limited use of the outdoor range by the hens, and the low availability of fibrous feedstuff in the most frequently used part of the range, together with the age of the animals, may explain why we found no effect of access to an outdoor range on the microbial community of these adult layers.

In order to sample commercial layer flocks, we collected cloacal swabs because the longitudinal follow-up required a rapid and minimally invasive sampling methodology, without sacrificing the birds. To ensure the cloacal swabs contained enough fecal material, the cloacal swabs were inserted deeply into the cloacal opening to enter the last part of the colon and the swabs were visually assessed prior to DNA extraction. Although research has shown that cloacal and fecal microbiota of chickens might not be an accurate representative of the cecal composition and are more variable (Williams and Athrey, 2020), it has also been shown that fecal samples are qualitatively similar to the cecal microbiota (Stanley et al., 2015) and non-shared taxa between cloacal and cecal samples accounted for a very low percentage of the diversity: 0.49% in one case (Andreani et al., 2020) and 0.75% in another (Stanley et al., 2015). Furthermore, it has been reported that cloacal swabs are similar to fecal samples (Videvall et al., 2018), and shifts in microbiota composition have been detected successfully using fecal samples (Oakley and Kogut, 2016; Jurburg et al., 2019). Therefore, we anticipated that major shifts in community composition would have been detected by our way of sampling. Nevertheless, future studies should carefully consider the trade-off between applicability of a sampling technique in commercial practice vs. the quality of the taken sample.

## Conclusion

In conclusion, our study gives insight into the temporal dynamics of the cloacal microbiota of adult layer flocks exposed to environmental change. We find that cloacal community composition in adult layers is rather stable, even after a sudden environmental change, illustrating the layers’ ability to maintain their own microbiota. Furthermore, we show the strong influence of poultry house on the microbiota composition of these layers, and that temporal dynamics are unique to each poultry house. Our study thus sheds light into the drivers of the poultry microbiota, and the stability of the adult chicken microbiota to environmental change, however our understanding of the temporal dynamics of adult animal microbiota remains limited. Future research should consider the influence of a host’s immediate environment (i.e., poultry house) and the animals’ previous exposure to environmental change (i.e., rearing farm). Furthermore, the stability of adult poultry microbiota should be tested in both healthy and diseased flocks, with shorter sampling intervals and larger sample sizes across multiple commercial flocks.

## Data Availability Statement

Availability of data and materials Raw sequence data were submitted into the Sequence Read Archive (SRA) at the NCBI under accession number PRJNA673103. The phyloseq object is available at 10.5281/zenodo.4155877.

## Ethics Statement

Ethical review and approval was not required for the animal study because only non-experimental procedures were used, which were minimally invasive and did not require ethical approval by the Dutch Central Authority for Scientific Procedures on Animals and the Animal Experiments Committee. No personal data of farmers was used and all data on the individual farms was anonymized. Written informed consent from the owners of the farms for participation was not required yet when the data was gathered in 2017 as the GDPR directive was only enforced in May 2018. Instead oral consent was given by the farmers.

## Author Contributions

RB, WdB, JS, AS, and JS initiated this project. RB, JS, WdB, PvH, SJ, AS, and FV contributed to the design of the experiment. JS performed sample collection and manuscript writing. JS, AB, and SJ did data processing and analysis. SJ, AS, FV, JS, RB, AB, PvH, and WdB contributed to the development of the manuscript by giving constructive feedback on the manuscript during its preparation. AS and SJ contributed equally and are both considered last author. All authors gave approval of the manuscript for publication.

## Conflict of Interest

The authors declare that the research was conducted in the absence of any commercial or financial relationships that could be construed as a potential conflict of interest.

## Supplementary Material

Supplementary Figure 1 Relative abundances of 10 most abundant phyla on average across all samples shown per timepoint and faceted per house.

Supplementary Figure 2 Relative abundances of 10 most abundant genera on average across all samples shown per timepoint and faceted per poultry house.

Supplementary Figure 3 Bray–Curtis distance based redundancy analysis (dbRDA) with model y=Poultry House+SamplingTime+HousingType+ SamplingTime:HousingType. Ellipses are drawn around each poultry house. A model with PoultryHouse+SamplingTime had the lowest AIC value (301.38) with constrained ordination and in this model, poultry house (*p* = 0.005, ANOVA) and time (*p* = 0.005, ANOVA) were both significant and the model explained 20.48% of the variation.

Supplementary Figure 4 Amplicon sequence variants (ASVs) that were significantly different over time between indoor and outdoor housed chickens as determined by DESeq2 analyses, grouped by genus on the *x*-axis and colored according to phylum membership.
