# Supplementary figures and images for: Temporal Dynamics of Cloacal Microbiota in Adult Laying Chickens With and Without Access to an Outdoor Range
Source: Front Microbiol. 2021 Jan 28;11:626713. doi: 10.3389/fmicb.2020.626713 (PMC7876281; doi:10.3389/fmicb.2020.626713)

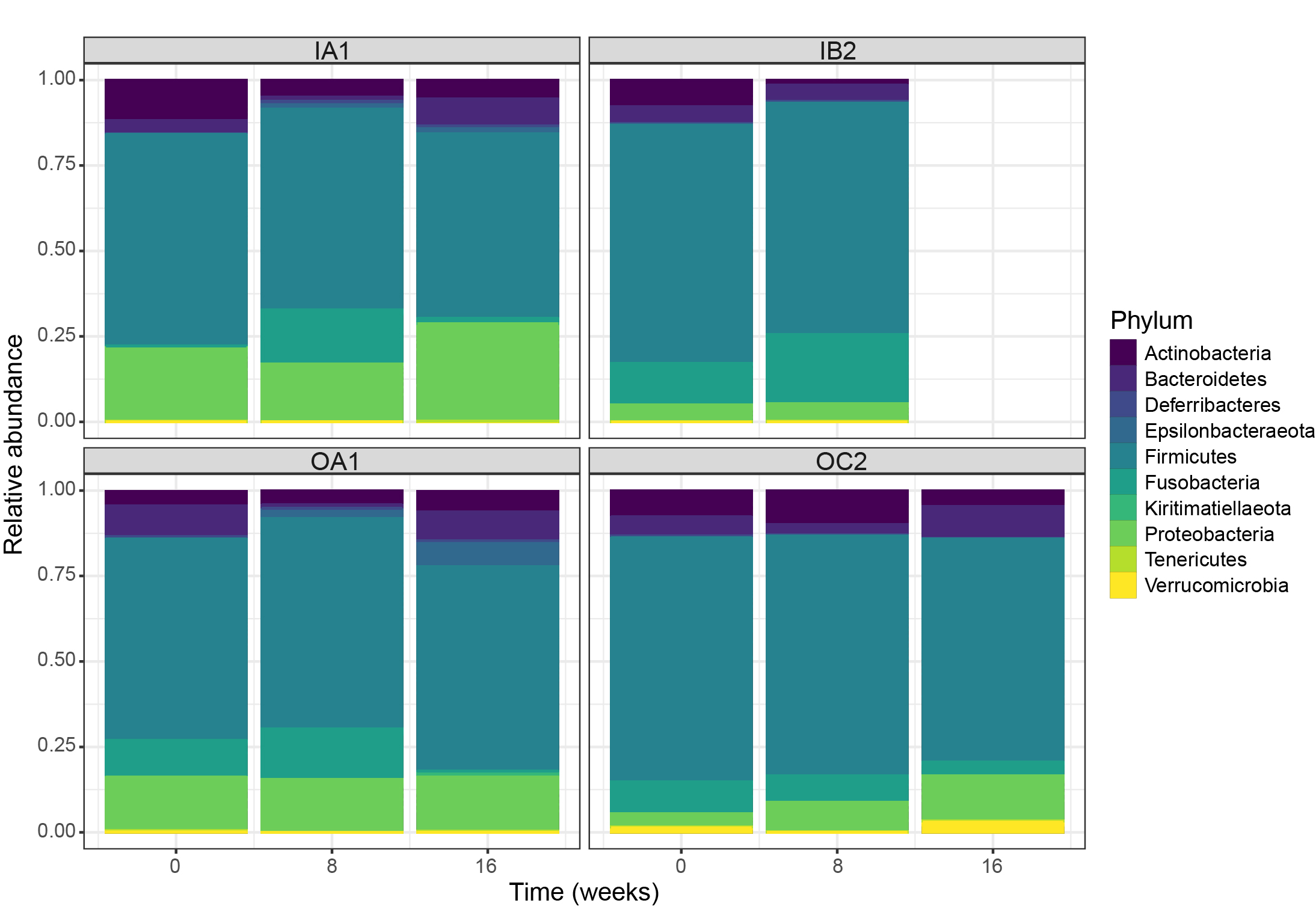

Supplement: Supplementary Figure 1 — Relative abundances of 10 most abundant phyla on average across all samples shown per timepoint and faceted per house. [file Image_1.JPEG]

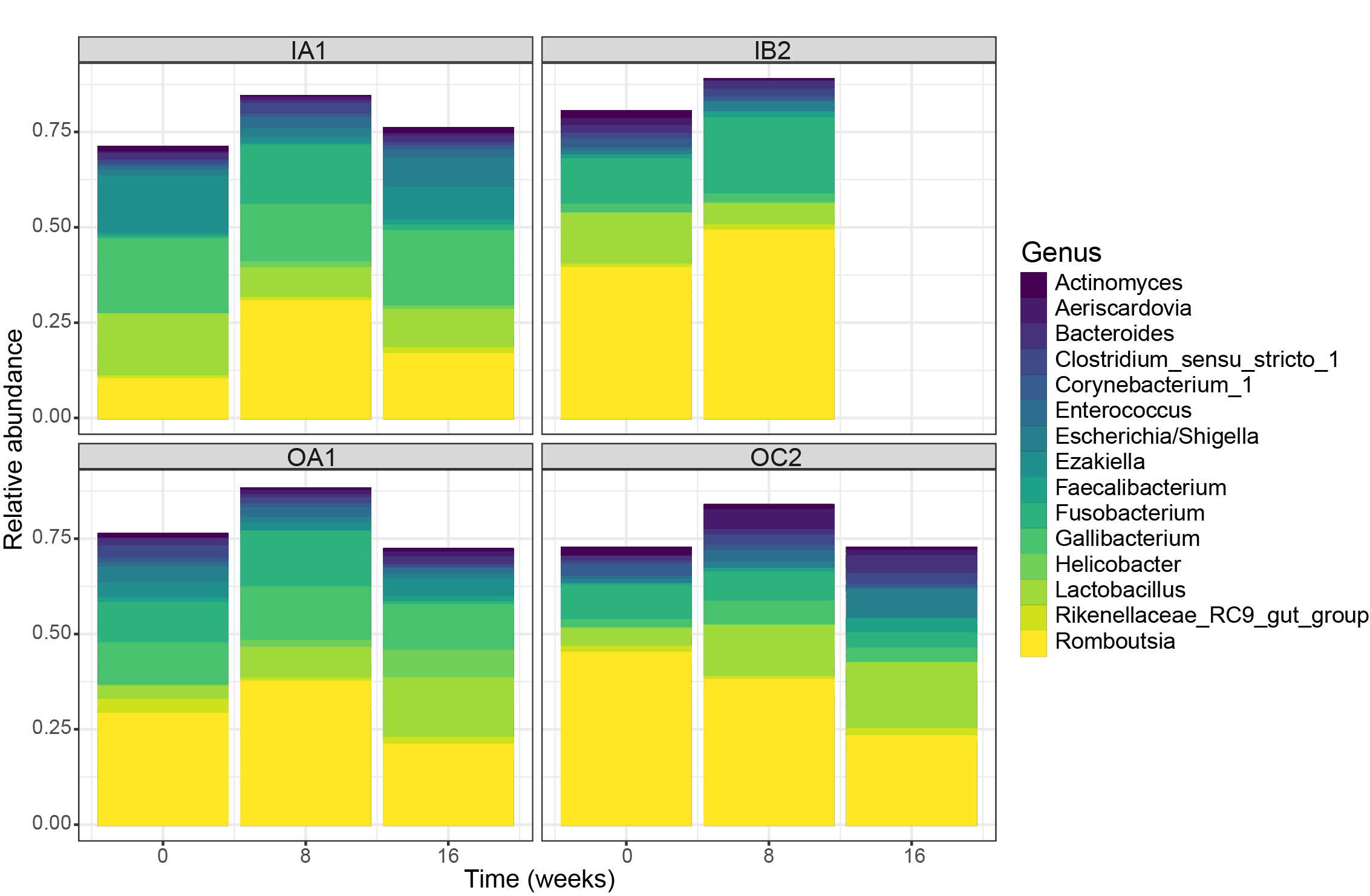

Supplement: Supplementary Figure 2 — Relative abundances of 10 most abundant genera on average across all samples shown per timepoint and faceted per poultry house. [file Image_2.JPEG]

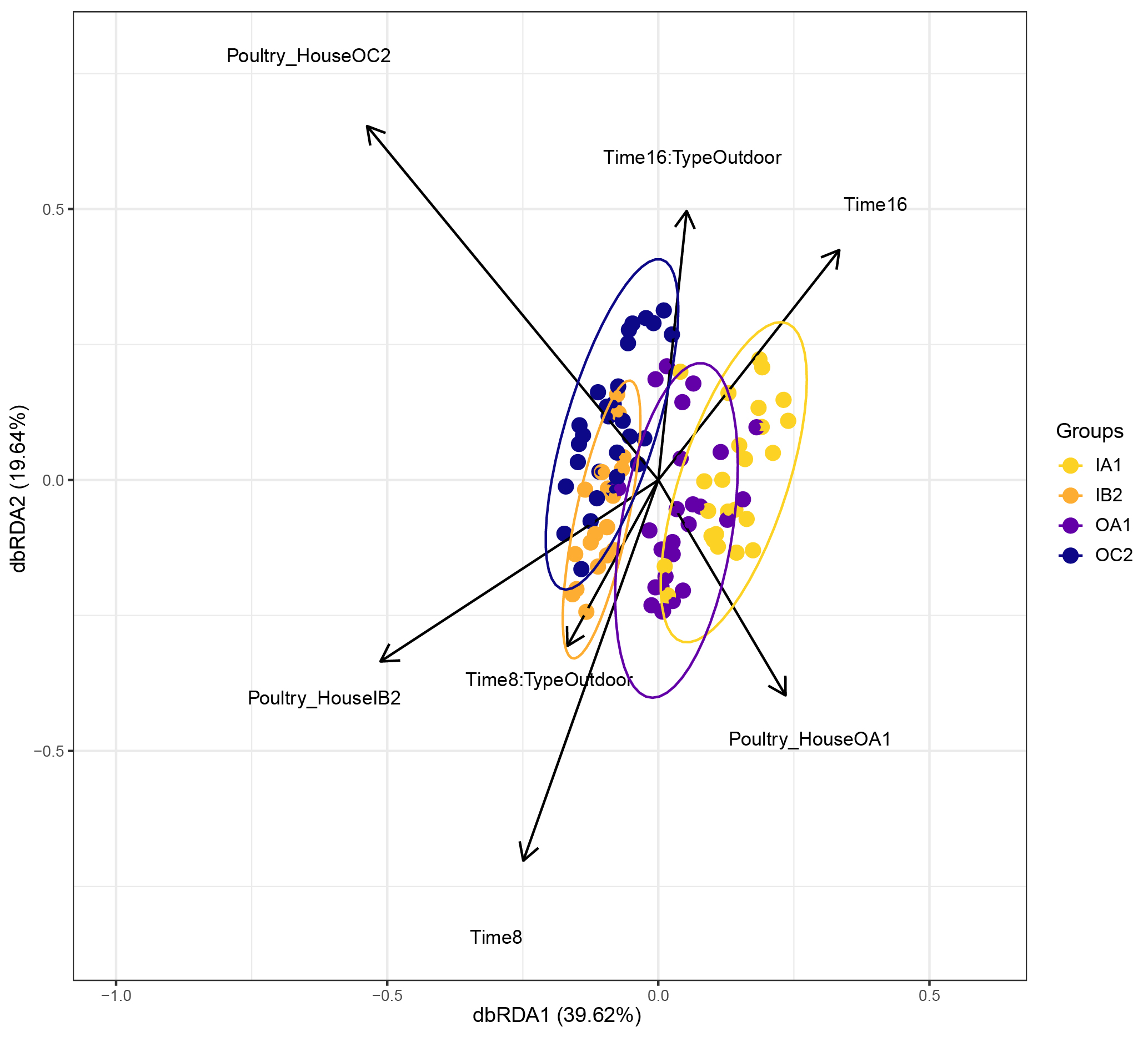

Supplement: Supplementary Figure 3 — Bray–Curtis distance based redundancy analysis (dbRDA) with model y=Poultry House+SamplingTime+HousingType+ SamplingTime:HousingType. Ellipses are drawn around each poultry house. A model with PoultryHouse+SamplingTime had the lowest AIC value (301.38) with constrained ordination and in this model, poultry house (p = 0.005, ANOVA) and time (p = 0.005, ANOVA) were both significant and the model explained 20.48% of the variation. [file Image_3.JPEG]

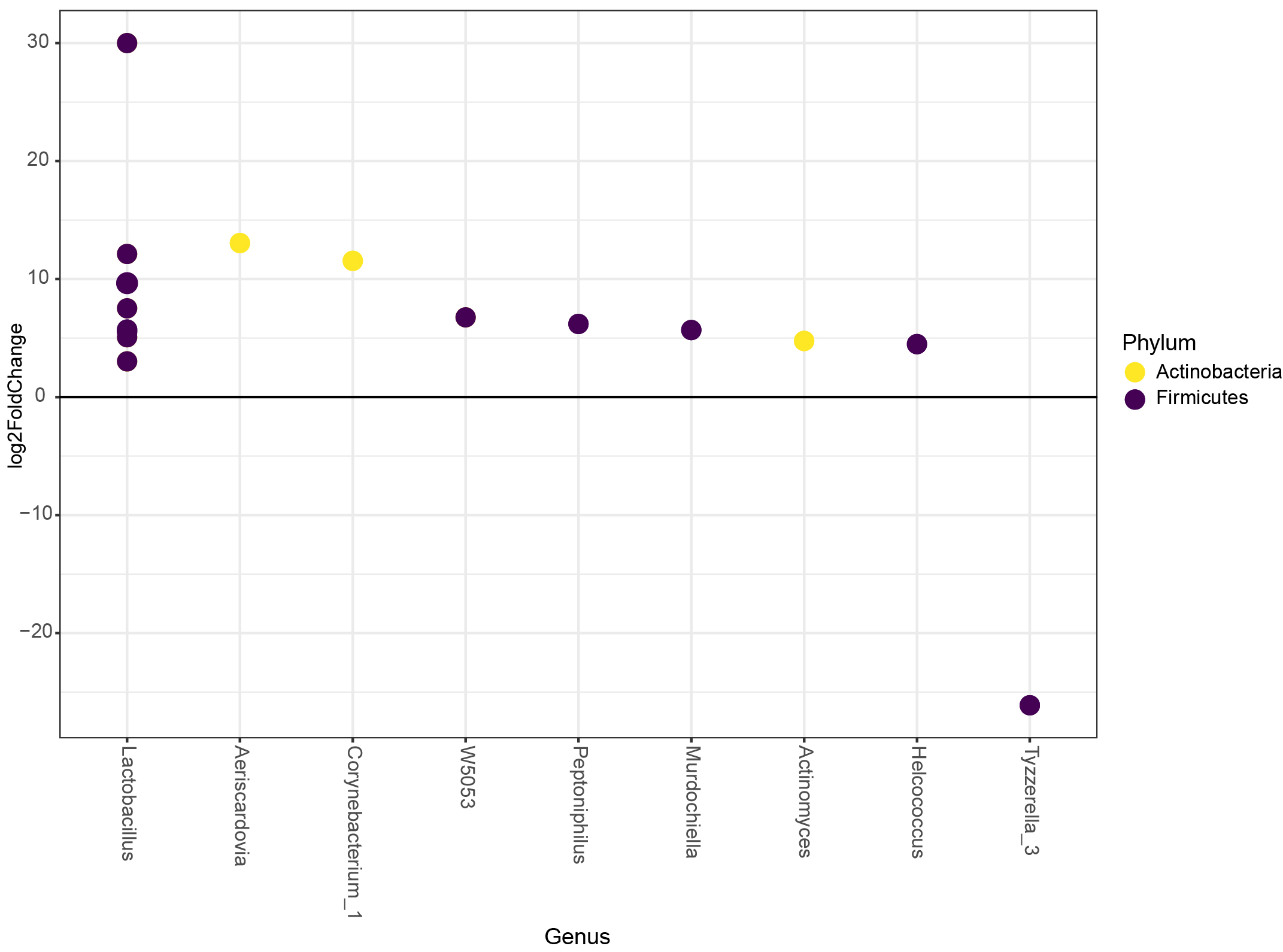

Supplement: Supplementary Figure 4 — Amplicon sequence variants (ASVs) that were significantly different over time between indoor and outdoor housed chickens as determined by DESeq2 analyses, grouped by genus on the x-axis and colored according to phylum membership. [file Image_4.JPEG]
